# Neural stem/progenitor cells react to non-glial cns neoplasms

**DOI:** 10.1186/s40064-015-0807-z

**Published:** 2015-02-03

**Authors:** Jack Griffin Campbell, Douglas C Miller, Diane D Cundiff, Qi Feng, N Scott Litofsky

**Affiliations:** Department of Surgery, Division of Neurological Surgery, The University of Missouri School of Medicine, Columbia, Missouri USA; Department of Pathology & Anatomical Sciences, The University of Missouri School of Medicine, M263 Medical Science Building, One Hospital Drive, Columbia, MO 65212 USA

**Keywords:** Sox2, Neural stem cells, Cerebral metastases, Brain lymphoma

## Abstract

It is well established that the normal human brain contains populations of neural stem/progenitor cells. Recent studies suggest that they migrate toward a variety of CNS tissue injuries. In an investigation of the potential role of neural stem cells in the pathogenesis of primary CNS lymphomas (NHL-CNS), we observed that neural stem/progenitor cells appeared to accumulate at the border of the tumors with the brain and in the advancing edge of the tumors, in a pattern similar to that seen with reactive gliosis. We identified neural stem/progenitor cells using standard immunohistochemical markers thereof, including CD133, nestin, Group II Beta-tubulin, Musashi1, and the transcription factor Sox2, in neurosurgically obtained specimens of NHL-CNS metastatic carcinoma , and metastatic melanoma . We had similar results with each of these markers but found that Sox2 antibodies provided the clearest and most robust labeling of the cells at the borders of these non-glial tumors. To exclude that the immunoreactive cells were actually neoplastic, double-label immunohistochemistry for Sox2 and CD20 (for NHL-CNS), Sox2 and cytokeratin (CAM5.2, for carcinomas), or Sox2 and HMB45 (for melanomas) showed that in each tumor type, Sox2-immunoreactive cells adjacent to and among the tumor cells were separate from neoplastic cells. Sox2/GFAP double-labeling revealed a consistent pattern of Sox2 immunopositivity both in reactive GFAP-immunopositive astrocytes and in GFAP-negative cells, at the interface of tumor and non-neoplastic brain. These results suggest that neural stem/progenitor cells migrate to non-glial neoplasms in the CNS, are a source of reactive astrocytes, and that Sox2 is a reliable immunohistochemical marker for these cells.

## Introduction

The cancer stem cell hypothesis has revolutionized studies of cancer biology, including those of primary brain tumors; it is now widely accepted that there is a stem cell component in these as in most cancers. However the related discovery of endogenous neural stem cells, specifically mostly in the subventricular zone, has led to the notion that neural stem cells might mediate responses to gliomas as well as representing a potentially transformable pool of cells that may be the source of such tumors. Endogenous neural stem/progenitor cells distinct from tumor cells have an innate tropism for both gliomas (Aboody et al. 2000) and non-glial CNS tumors metastatic to the CNS (Aboody et al. 2006) and also secrete antitumorigenic factors such as vanilloids and BMP-7 (Chirasani et al. 2010; Stock et al 2012). Macas et al. (2014) recently demonstrated that normal neural progenitor cells, characterized by expression of polysialated neural cell adhesion molecule (PSA-NCAM) and BIII-tubulin populate the non-neoplastic but reactive brain tissue adjacent to both high-grade astrocytomas and non-neural cerebral metastases. The migration of neural stem cells into the region of a tumor in a mouse model was shown to be mediated by vascular endothelial growth factor (VEGF) secreted by tumor cells signaling through VEGF Receptor 2.

Accurate characterization of the phenotype and function of endogenous neural stem/progenitor cells has clinical applications given that intracerebral injection of engineered neural stem/progenitor cells is being explored as treatment of neoplasms in the brain (Aboody et al. 2006; Li et al. 2005; Rath et al. 2009; Sanson et al. 2002). Proposed tumoricidal mechanisms include gene therapy using a viral-mediated HSV/tk/GCV process (Li et al. 2005; Rath et al. 2009), or with cytosine deaminase for local generation of 5-fluorouracil at the site of tumor (Yang et al. 2011). There is an ongoing clinical trial for recurrent high-grade gliomas with the latter strategy (ClinicalTrials.gov; Identifier: NCT01172964).

Neural stem cells and progenitor cells have been shown to be selectively labeled by a variety of immunohistochemical markers, including the fetal brain intermediate filament protein nestin (Sugawara et al. 2002), the early neuronal differentiation marker group II beta-tubulin (Sugita et al. 2005), the RNA-binding protein Musashi1 (Okano et al. 2005), the transcription factor Sox2 (Pevny and Nicolis 2010), and the stem cell antigen CD133 (Li 2013). Sox2is an HMG box transcription factor with a documented role in embryogenesis and maintainance of pluripotent stem cells (Rath et al. 2009). Sox2 has been implicated in the oncogenesis of glial neoplasms in the brain (Alonso et al. 2011; Annovazzi et al. 2011; Guo et al. 2011; Schmitz et al. 2007), and has also been detected in cells of epithelial and mesenchymal tumors (Basu-Roy et al. 2011; Girouard et al. 2012; Mimeault and Batra 2011; Nakatsugawa et al. 2011), but is also a marker of neural stem cells where its function appears to be maintenance of pluripotency (Pevny and Nicolis 2010). In addition to the endogenous role of Sox2 in the non-pathologic state, populations of Sox2-immunopositive cells have been detected near sites of injury in the CNS in mice including lesions of demyelinating disease, hypoglossal nerve avulsion, and penetrating stab wounds (Fagerlund et al. 2011; Magnus et al. 2007; Rasmussen et al. 2011). In human brain following subarachnoid hemorrhage excised cortex had upregulated mRNA expression of Sox2 and Musashi2 as evaluated by PCR, in tandem with other markers of proliferation; this was matched by immunohistochemical detection of Musashi2 in tandem with the proliferation marker Ki67 (Sgubin et al. 2007) . In this study, we show that neural stem/progenitor cells labeled by Sox2, Musashi1, nestin, CD133 or beta-tubulin antibodies accumulate at the borders of primary CNS B cell non-Hodkin lymphomas (NHL-CNS) and of metastatic non-neural neoplasms in the brain.

## Materials and methods

### Tissue preparation

All tissue samples were surgically removed, formalin-fixed, and paraffin embedded between 2008 and 2012, and were retrieved from the archives of the Department of Pathology of the University of Missouri Hospitals and Clinics with appropriate IRB approval with waiver of informed consent. New sections cut from the paraffin blocks at 5 μm thickness were used for immunohistochemical stains (IHC).

The cases examined included 5 NHL-CNS, and 37 metastatic tumors in the brain (26 carcinomas, 11 melanomas).

### Immunohistochemistry

Antibodies:

IHC stains were done with antibodies to nestin (mouse monoclonal, dilution 1:400; catalog no. MA1-46100, Pierce, Rockford, IL), Musashi1 (rabbit polyclonal, dilution 1:100, catalog no. PA1-30152, Pierce), beta-tubulin (rabbit polyclonal, dilution 1:100, catalog no. PA1-41331, Pierce), CD133 (mouse monoclonal, dilution 1:2000,catalog no. MA5-15875, Pierce), Sox2 (Goat polyclonal, dilution 1:100; catalog No. AR2018, R&D Systems, Minneapolis, MN), GFAP (Rabbit polyclonal, dilution 1:200; catalog No. 16825-1-AP, Proteintech Group, Chicago, IL), CD20 (Rabbit polyclonal, dilution ready-to-use; catalog No. PA1-37311, Pierce/Thermo-Fisher, Rockford, IL), HMB45 (Mouse monoclonal, dilution 1:50; catalog No. M0634, DAKO, Carpinteria, CA), and low molecular weight cytokeratins CAM5.2 (Mouse monoclonal, dilution ready-to-use; catalog No. 349205, Becton Dickinson, Franklin Lakes, NJ).

Initial IHC for nestin, Musashi1, beta-tubulin, CD133, and Sox2 was performed on 5 NHL-CNS, and on 5 glioblastomas and 5 cases of systemic NHL (the latter two as controls). Subsequently, based on the performance of the various antibodies to stem/progenitor cell markers, we concentrated on immunostains for Sox2 on 26 carcinomas metastatic to brain, and 11 melanomas metastatic to brain. IHC was carried out with a standard protocol involving heat-induced epitope retrieval with Target Retrieval pH6 (S1700; DAKO, Carpinteria, CA). Sox2 antibody incubation was performed for 1 hour at room temperature and the antibody was detected using a biotinylated link universal and streptavidin-HRP system (K0690; DAKO). One case of carcinoma and three of melanoma metastatic to brain were excluded from further study due to paucity of non-neoplastic brain adjacent to tumor in the tissue blocks selected for this study.

Double-label IHC was performed with Sox2 and CD20 antibodies on five cases of CNS lymphoma; Sox2 and HMB45 on 6 melanomas metastatic to brain; and Sox2 and CAM5.2 on 9 carcinomas metastatic to brain which clearly lacked Sox2-immunopositive tumor cells. Additionally, double-staining with Sox2 and GFAP was performed on all cases of NHL-CNS and those metastatic tumors for which the other double stains were done. The procedure for staining with Sox2 was the same as that described above except that the dual endogenous enzyme block was performed before treating with the primary Sox2 antibody. After treating with the DAB+ substrate chromagen system, the slides were rinsed with distilled water. Three minutes of doublestain block was followed by a 30-minute, 20-minute, or 10-minute incubation with anti-CD20, CAM5.2/HMB45, and GFAP, respectively. Visualization of the second antibody was performed with an Envision Alkaline Phosphatase Permanent Red System (K5361; DAKO) and the sections were then counterstained with Mayer’s hematoxylin. Following counterstaining, slides were dried and coverslipped with Aqua-Mount (13800; Thermo Scientific).

Following the initial interpretation of the results, we concluded that distinction between tumor cells and adjacent non-pathologic brain did not depend on immunohistochemical labeling but was evident based on architecture and cytology. Therefore we double-labeled 10 additional cases of carcinoma metastatic to brain which had extensive Sox2 immunopositivity in the tumor cells; these cases were examined with Sox2/GFAP using the above procedure.

## Results

**Figure 1:**
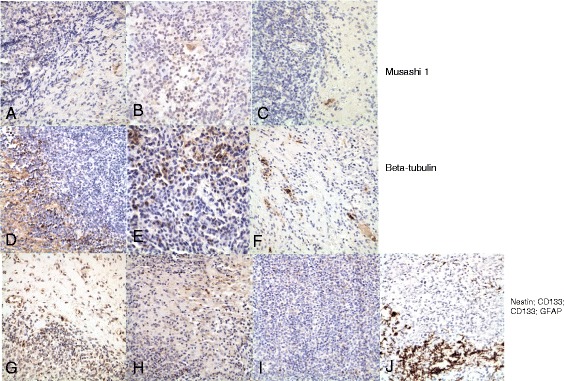
**Immunoreactivity for neural stem/progenitor cell markers in cases of primary CNS lymphoma (NHL-CNS). A-C)** are sections of 3 different cases of NHL-CNS immunostained for Musashi-1. There are rare immunopositive cells within the tumor or at the edges of the tumor in the adjacent gliotic brain. **D-F)** are sections of the same tumors immunostained for group II beta-tubulin. There is some background immunopositivity in the brain of neural processes, but cytoplasmic immunopositivity is seen in only in relatively rare cells along the edges of the tumors and, less frequently, within the tumors. **G)** Immunostain for nestin in a section from a case of NHL-CNS. There are some nestin-immunopositive cells along the border between the brain and the lymphoma, transgressing a short distance into the tumor between the lymphoma cells. **H** and **I**) Immunostains for CD133 in two cases of NHL-CNS. In **(H)** there are immunopositive cells in the brain between two portions of tumor; in **(I)** there are rare immunopositive cells within the tumor cell aggregates. For comparison to all of these, **(J)** is an immunostain for GFAP in one of these cases, demonstrating maximal gliosis along the border between the tumor and the brain with a few entrapped GFAP-immunopositive astrocytes among the tumor cells deeper into the neoplastic aggregate. All photomicrographs at original magnification of 400x.

**Figure 2:**
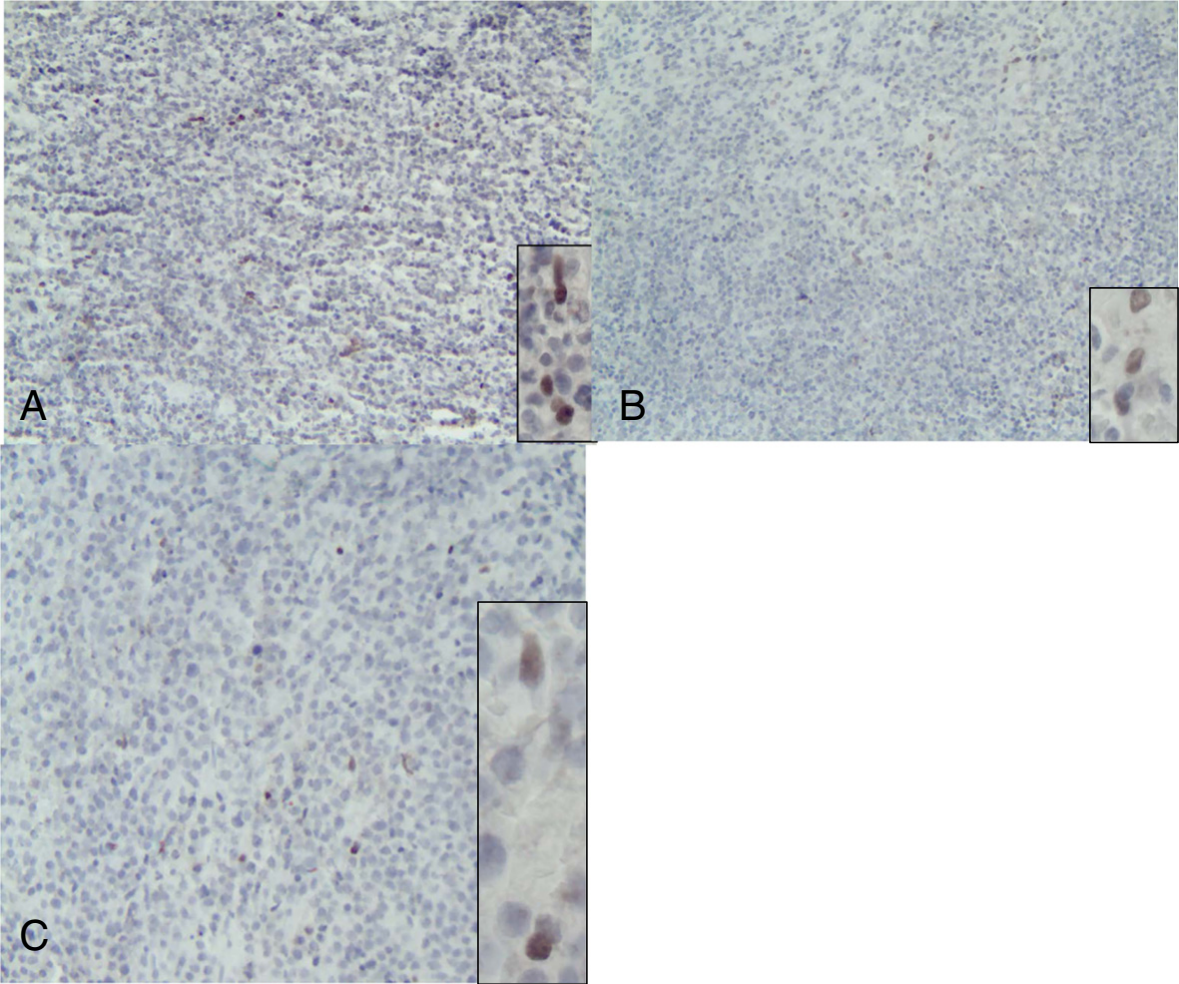
**Immunostains of NHL-CNS with antibody to Sox2.** Each photomicrograph **A-C** shows scattered sparse cells with Sox2-immunopositive nuclei among the tumor cells. These were also present at the borders of the tumor with the adjacent brain, and were more numerous in the gliotic brain than in the densely cellular portions of the tumors. Each photomicrograph at original magnification of 100x; insets showing immunopositive nuclei original magnifications at 400x.

Initial immunostains for stem/progenitor cell markers in sections of primary CNS lymphomas all showed variably sparse immunoreactive cells at the interface between groups of tumor cells and the adjacent (gliotic) brain (Figure 1 all but Sox2; Figure 2 Sox2). Most of the cells immunopositive for each of these markers were in the peripheral portions of the tumor, and not deep in tumor masses, or were in the gliotic brain bordering the tumor. Some of the cells with immunoreactivity to Musashi1 (Figure 1A-C) were somewhat larger and resembled reactive astrocytes, as did some of the Sox2-immunopositive cells (Figure 2); cells with immunoreactivity for nestin (Figure 1G), CD133 (Figure 1H,I), and beta-tubulin(Figure 1D-F) were in general small. The pattern of distribution of these cells was similar to, but less dense than, that of reactive astrocytes in the same tumors as demonstrated with GFAP immunostains (Figure 1J).

**Figure 3:**
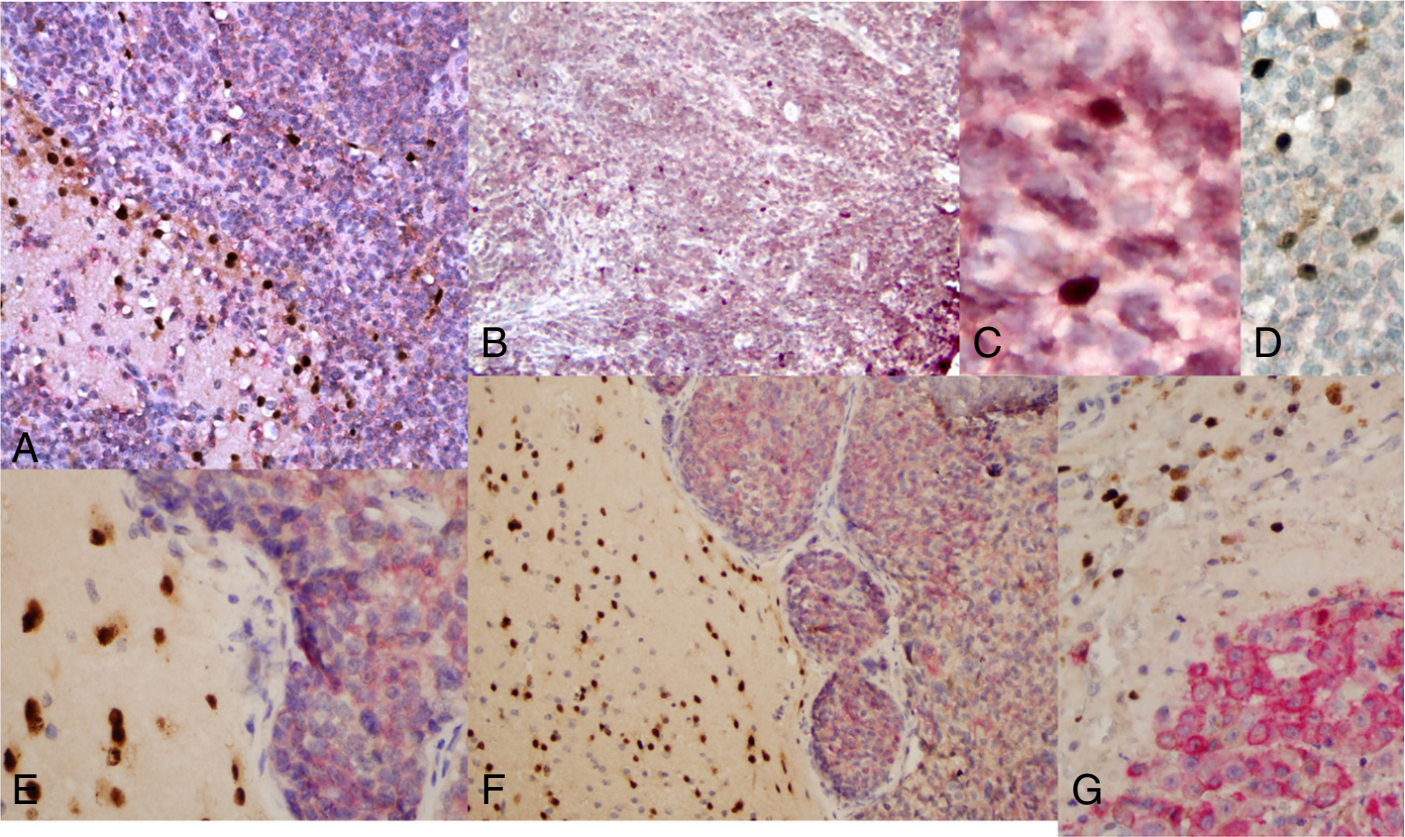
**Double-label immunostains for Sox2 (brown, nuclear) and tumor-specific markers. A)** NHL-CNS labeled for CD20 (red, cytoplasmic/membranous) along with nuclear Sox2. The Sox2 immunoreactive nuclei are most numerous in the brain next to the tumor and at the interface with the tumor. No cells are double-positive for CD20 and Sox2. Original magnification 200x. **B)** Another case of NHL-CNS double-labeled for CD20 and Sox2. The scattered Sox2-immunopositive nuclei are each without associated cytoplasmic/membranous CD20 immunoreactivity. Original magnification 100x. **C)** High magnification of Sox2-immunopositive nuclei within the NHL-CNS of **(B)**. The absence of CD20-immunopositivity around the Sox2-immunopositive (brown) nuclei is more obvious. Original magnification 400x. **D)** Another case of NHL-CNS with CD20-negative Sox2-immunopositive cells, and CD20-immunopositive Sox2-negative tumor cells. Original magnification 400x. **E)** Metastatic carcinoma in brain, double-labeled for Sox2 (brown) and for cytokeratin (CK) (antibody CAM5.2). The tumor cells have CK-immunoreactive cytoplasm with no nuclear Sox2 immunopositivity; the adjacent brain has many cells with Sox2-immunopositive nuclei without cytoplasmic CK, with a few of these entrapped between tumor cells. Original magnification 200x. **F)** Another metastatic carcinoma in the brain, double-labled for Sox2 (brown) and for cytokeratin (antibody CAM5.2). As in “**E**”, the tumor cells have CK-positive cytoplasm with no nuclear Sox2 immunoreactivity, while the adjacent brain has cells with Sox2-immunopositive nuclei without any cytoplasmic CK immunoreactivity. Original magnification 200x. **G)** A metastatic melanoma immunostained for HMB45 (red) and for Sox2 (brown). The Sox2-immunopositive nuclei are mostly within the adjacent brain at the edge of the tumor, and all lack HMB45 cytoplasmic immunoreactivity; the melanoma cells are HMB-45 immunopositive but do not have any Sox2 nuclear immunoreactivity. Original magnification 400x.

Sox2 IHC of 5 NHL-CNS with adjacent brain revealed Sox2-immunopositive cells in every case, also primarily at the interface of the tumor with the adjacent non-neoplastic brain but also with occasional cells within the masses of the tumors (Figures 2 and 3A). Similarly, Sox2 IHC of 25 metastatic carcinoma and 10 metastatic melanomas in brain showed the same pattern, except that 16 and 2 cases of carcinoma and melanoma, respectively, had abundant Sox2-immunopositive tumor cells, consistent with previous literature (Basu-Roy et al. 2011; Girouard et al. 2012; Mimeault and Batra 2011; Nakatsugawa et al. 2011) (not shown). In such cases, double-labeling with Sox2 and a tumor marker would be superfluous and was not performed.

Both because we found Sox2 immunostains more robust and reliable than those for Musashi1 or CD133, and more specific than those for nestin (which also, for example, are immunopositive in blood vessel cells as well) or beta-tubulin, and because Sox2 is a nuclear antigen suitable for double-label immunoperoxidase stains with other cytoplasmic markers, we did further analyses only with Sox2 as a marker of putative neural stem/progenitor cells. Double-staining was performed with both Sox2 and a second tumor-specific marker (CD20 for NHL-CNS; CAM5.2 for carcinomas; HMB45 for melanoma) on the remaining 20 metastatic cases to determine whether the Sox2-immunopositive cells might be part of the neoplastic cell population (Figure 3). None of the 20 cases established not to have predominantly Sox2 immunopositive tumor cells had any co-localization of Sox2 and the tumor-specific marker*, ie* no lymphoma cells with CD20 immunoreactivity were also Sox2-immunopositive, no carcinoma cells with CAM5.2 immunopositive cytoplasm had any nuclear Sox2 immunoreactivity, and no melanoma cells with HMB45 cytoplasmic immunopositivity had Sox2-immunoreactive nuclei (Figure 3). In these sections of double-stained tumors and brain, the Sox2-immunopositive cells were in the gliotic brain tissue adjacent to these non-neural neoplasms, with the greatest concentration close to the tumor edges and in the outer portions of the tumors (particularly the lymphomas) with fewer in brain at a distance from the tumors.

**Figure 4:**
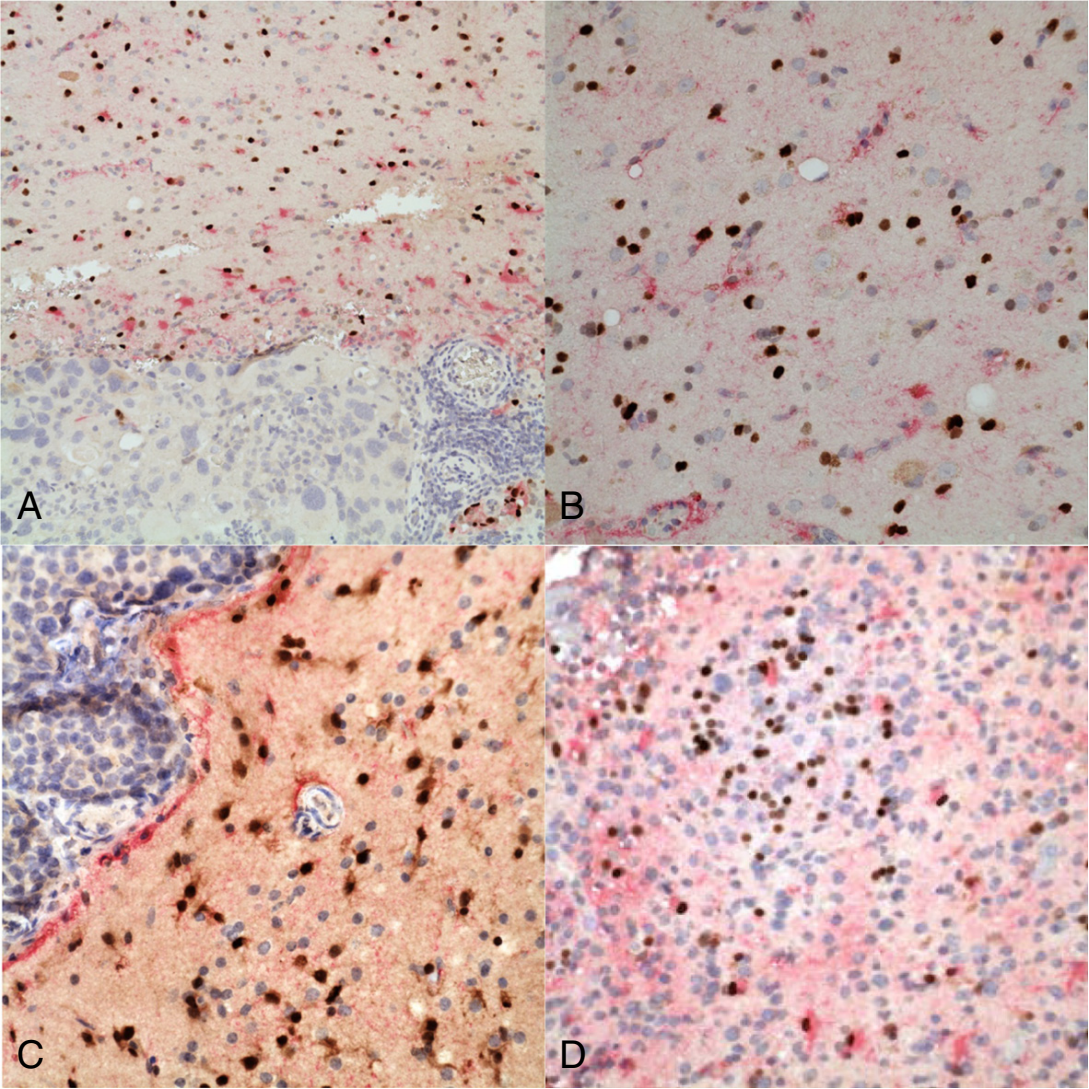
**Double-label immunostains for Sox2 (brown, nuclear) and GFAP (red, cytoplasmic) in non-neural CNS tumors. A)** Metastatic carcinoma in brain. There is reactive gliosis as indicated by the GFAP-positive cells (with red cytoplasm) at the edges of the neoplasm. Some of these reactive astrocytes have Sox2-immunopositive (brown) nuclei; others do not. There are rare entrapped astrocytes and Sox2-positive nuclei within the tumor. Original magnification 100x. **B)** Gliotic brain next to a metastatic carcinoma. The three populations of cells described in the text are apparent here: cells with Sox2-immunopositive nuclei without GFAP-immunopositive cytoplasm; reactive astrocytes with GFAP-immunopositive cytoplasm and Sox2-negative nuclei; and cells with double-labeling for both GFAP and Sox2. Rare cells with brown cytoplasm contain melanin and are tumor cells. Original magnification 400x. **C)** Edge of a metastatic carcinoma in brain. Again there are variably Sox2 and GFAP immunopositive and negative cells as described previously. Original magnification 400x. **D)** NHL-CNS with double labeling. There are fairly numerous Sox2-immunopositive (brown) nuclei here near the edge of the tumor close to brain (not seen in this image), and a minority of those have associated GFAP-immunopositive (red) cytoplasm. Some reactive astrocytes with GFAP immunoreactivity but no Sox2-immunopositivity are also entrapped among the neoplastic lymphoid cells. Original magnification 400x.

Clearly, the brain adjacent to these neoplastic processes has reactive gliosis. This result raised the question as to whether in these situations Sox2 represented a marker of mature reactive astrocytes and not reactive stem/progenitor cells. We investigated this question using double-labeling of all of the tumor samples previously used for double-labeling with Sox2 and tumor-specific antibodies with IHC for Sox2 and GFAP (Figure 4). These double-labeling experiments identified three different immunophenotypic cell populations, all in the gliotic brain adjacent to the neoplasms. These were: (Aboody et al. 2000) astrocytes with Sox2-immunopositive nuclei and GFAP-immunopositive cytoplasm; (Aboody et al. 2006) astrocytes with GFAP-immunopositive cytoplasm without Sox2 immunoreactivity in their nuclei; and (Alonso et al. 2011) Sox2-immunopositive nuclei without any GFAP immunopositive cytoplasm (Figure 4). These results suggest that these non-neural tumors evoke a response by native neural stem/progenitor cells, which migrate into the zone adjacent to the tumor and among the tumor cells at the edges of the tumors, and which then can mature into reactive astrocytes.

The consistent pattern of cells immunopositive for neural stem cell or progenitor cell markers at the border of tumor and normal brain and the distinction between these cells and tumor cells achieved with double-labeling for Sox2 and a tumor-specific marker prompted further investigation into cases with Sox2-immunopositive tumor cells. Thus, 10 cases of carcinoma metastasis with uniformly Sox2-immunopositive nuclei for which double-labeling for Sox2 and a tumor-specific marker was not performed were used for Sox2/GFAP double-labeling. The Sox2/GFAP double-labeling in these cases was consistent with the previous cases, *ie* in the brain adjacent to the tumor there were many cells with nuclear Sox2 immunopositivity which lacked cytoplasmic GFAP immunoreactivity, in addition to other cells which were double-labeled and a third population of cells which had only GFAP without nuclear Sox2 immunoreactivity. One such case is illustrated in Figure 4*n*. Thus we analyzed 30 cases by double labeling for Sox2 and GFAP, all of which showed the consistent pattern of Sox2-immonopositivity described and illustrated here in Figures 2 and 3.

## Discussion

Sox2 is an HMG box transcription factor, one of the Yamanaka factors which exhibit characteristics of a master regulator transcription factor. It has a documented role in embryogenesis, including maintaining pluripotency of embryonic stem cells (Rizzino 2009) and self-renewal and multipotentiality of mesenchymal stem cells. Loss of Sox2 expression has been shown to inhibit stem cell multipotentiality and induce cell differentiation (Yi et al. 2012). Sox2 has also been shown to be effective in stimulating de-differentiation of somatic cells into pluripotent stem cells (Yoon et al. 2011). Interestingly, it appears the functions of Sox2 in non-pathologic stem cells of the body are likely exploited during oncogenesis. Among cancers Sox2 has primarily been studied for a role in oncogenesis of gliomas and it is expressed in many gliomas, most notably glioblastomas (Annovazzi et al. 2011; Guo et al. 2011; Schmitz et al. 2007). Levels of Sox2 upregulation appear to correlate with aggressive tumor behavior (Guo et al. 2011) and knockdown of Sox2 in brain tumor stem cells results in a loss of self-renewal, invasion, and migration of these cells (Alonso et al. 2011). Immunopositivity for Sox2 has been documented in examples of various other cancers including, but not limited to, lung adenocarcinoma (Nakatsugawa et al. 2011), melanoma (Girouard et al. 2012), breast carcinomas (Leis et al. 2012), and prostate carcinoma (Mimeault and Batra 2011). Our study is consistent with a wide variation of Sox2 expression;, some metastatic tumors had widespread Sox2 nuclear immunoreactivity; others, however, had none. Specifically, Sox2 was detected in individual cases of melanoma, lung small cell and adenocarcinoma, clear cell renal cell carcinoma, squamous cell carcinoma, and poorly-differentiated carcinomas of unproven origin.

With regard to the role of Sox2 in normal neural stem cells, (Pevny and Nicolis 2010) outlined five major observations. 1) Sox2 is expressed in neural stem cells as defined by the abilities of self-renewal and differentiation into neurons, astroglia, and oligodendroglia. 2) Sox2 maintains properties of neural stem cells, including pluripotency. 3) Maintenance of neural stem cells in the brain and eye requires Sox2 expression. 4) Sox2 is essential for neural differentiation in the brain and eye. 5) Sox2 function is both dose and context-dependent. This study expands upon these functions of Sox2 in neural stem cells to include a role in reactive neural stem/progenitor cells accumulating at the interface of brain and non-glial neoplasms of the CNS. Though the migration of these cells has been widely reported, to our knowledge, the detection of Sox2-immunopositivity in this cell population is a novel finding. This is also the first study detecting such a neural stem/progenitor cell response to neoplasms in the brain of specifically hematopoietic origin. Our results suggest that these migrating stem/progenitor cells mature into reactive astrocytes, transitioning through a stage in which they maintain Sox2 nuclear immunoreactivity while expressing cytoplasmic GFAP, and then go on to lose the Sox2 expression as mature reactive astrocytes.

Though this is the first report of a Sox2 as a marker of neural stem cell response to brain neoplasia, mice models have previously detected Sox2-positive cells near other sources of CNS injury including penetrating stab wounds and hypoglossal nerve avulsion (Fagerlund et al. 2011; Magnus et al. 2007). In the former, most Sox2-immunopositive cells failed to co-express GFAP, consistent with our finding that only a minority of the reactive cells double-labeled for both GFAP and Sox2. In an animal model of multiple sclerosis Rasmussen *et al.* (Rasmussen et al. 2011) used BrdU/Sox2 double-labeling to mark neural stem/progenitor cells, and found BrdU/Sox2 positivity near areas of injury in chronic demyelinating disease. They also noted that Sox2 expression was highest in the acute phase of demyelination with a decrease in periventricular Sox2-immunopositive cells in the chronic phase. This suggests that depletion of neural stem cells may contribute to the progression of demyelinating diseases.

Recently, (Macas et al. 2014) demonstrated similar findings to ours: they showed that neural stem/progenitor cells near brain tumors (gliomas and metastatic tumors) were genetically distinct from the tumor cells. For example, reactive stem/progenitor cells lacked Her2 amplification whereas adjacent breast carcinoma cells had such amplification. While their methods were quite different they reached the same conclusions we have, that neural stem/progenitor cells migrate toward the edges of tumors in reaction to them and are a separate non-neoplastic population from the cells of the tumors.

Also, (Sgubin et al. 2007) showed up-regulation of expression a variety of stem/progenitor cell markers, including Sox2 and Musashi2, in brain tissue from 4 human patients with subarachnoid hemorrhages from ruptured aneurysms, and, using immunostains, demonstrated a correlation between Musashi2-immunopositive cells and Ki67-immunopositive cells. Their findings also suggest that neural stem cells and progenitor cells respond to brain injuries by migration to as well as proliferation in the site(s) of injury.

There is evidence that reactive glia possess the capability for de-differentiation, however the degree to which glia undergo this phenomenon under physiologic conditions is incompletely understood (Costa et al. 2010; Steindler and Laywell 2003; Yang et al. 2011). Therefore, a possibility that emerges from these results is that the population of cells expressing stem/progenitor cell markers are reactive astrocytes undergoing de-differentiation and thus have gained Sox2 nuclear immunoreactivity or CD133, Musashi1, nestin, or beta-tubulin cytoplasmic immunoreactivity, but are not derived from neural stem/progenitor cells. In other words, the cell population might be locally generated rather than represent a migration from a neural stem cell niche. Though Sox2 is not a classic marker for astrocytes, Sox2 immunopositivity in parenchymal astrocytes expressing GFAP has been documented (Komitova and Eriksson 2004). Our results are at least now wholly inconsistent with this possibility, however the paucity of Sox2-immunopositive cells which co-express GFAP relative to cells singularly expressing Sox2 is striking and favors the hypothesis that these are cells migrating in from a stem/progenitor cell niche and then differentiating to astrocytes, rather than the reverse. If Sox2-positive/GFAP-negative cells originate from astrocytes, it would appear unlikely for the majority of these cells to lack GFAP, an established and reliable marker of astrocytes. Furthermore, the extensive characterization of migrating neural progenitor cells by (Macas et al. 2014) establishes a known population of neural progenitor cells in the same “geographic” pattern as that observed in this study. While it is theoretically possible that the neural stem cell marker Sox2 could be expressed in two separate cell populations (neural progenitor cells and reactive astrocytes) which share the same anatomic location with respect to brain neoplasms, it seems unlikely.

## Conclusions

This study emphasizes the utility of Sox2 as a marker of neural stem/progenitor cells, and supports the hypothesis that these cells respond to non-glial neoplasms of the CNS of epithelial and hematopoietic origin. At the site of non-glial CNS neoplasms, these cells mature into reactive astrocytes and thus serve as the origin of gliosis. As the therapeutic use and understanding of neural stem/progenitor cells increases in scope, detailed characterization of the phenotype and physiology of neural stem/progenitor cells will hopefully lead to novel treatments and an increased survival, especially in cases of high-grade gliomas, which have to this point, been refractory to treatment. Furthermore, this study demonstrates that the detection of neural stem/progenitor cells can be performed with a marker, Sox2, readily available in many, if not most, surgical pathology departments, using IHC, which has obviously become an essential tool of all practicing surgical pathologists.
